# Aurantoside J: a New Tetramic Acid Glycoside from *Theonella swinhoei*. Insights into the Antifungal Potential of Aurantosides 

**DOI:** 10.3390/md9122809

**Published:** 2011-12-20

**Authors:** Rihab F. Angawi, Giorgio Bavestrello, Barbara Calcinai, Henny Adeleida Dien, Giovanna Donnarumma, Maria Antonietta Tufano, Iole Paoletti, Elena Grimaldi, Giuseppina Chianese, Ernesto Fattorusso, Orazio Taglialatela-Scafati

**Affiliations:** 1 The NeaNat Group, Department of Natural Compounds Chemistry, University of Naples “Federico II”, Via D. Montesano 49, 80131 Napoli, Italy; Email: rangawi@kau.edu.sa (R.F.A.); g.chianese@unina.it (G.C.); fattoru@unina.it (E.F.); 2 Department of Marine Sciences, Polytechnic University of Marche, Via Brecce Bianche, 60131 Ancona, Italy; Email: g.bavestrello@univpm.it (G.B.); b.calcinai@univpm.it (B.C.); 3 Faculty of Fishery and Marine Science, Sam Ratulangi University, Manado, Indonesia; Email: hennydien@yahoo.com; 4 Department of Experimental Medicine, Section of Microbiology and Clinical Microbiology, Second University of Naples, Via Costantinopoli, 16-80138 Naples, Italy; Email: giovanna.donnarumma@unina2.it (G.D.); mariaan.tufano@unina2.it (M.A.T.); iolepaoletti@libero.it (I.P.); ele.grimaldi@virgilio.it (E.G.)

**Keywords:** *Theonella swhinoei*, aurantosides, *N*-glycosides, antifungal activity

## Abstract

The chemical investigation of an Indonesian specimen of *Theonella swinhoei* afforded four aurantosides, one of which, aurantoside J (**5**), is a new compound. The structure of this metabolite, exhibiting the unprecedented *N*-α-glycosidic linkage between the pentose and the tetramate units, has been determined through detailed spectroscopic analysis. The four obtained aurantosides have been tested against five fungal strains (four *Candida* and one *Fusarium*) responsible of invasive infections in immuno-compromised patients. The non-cytotoxic aurantoside I (**4**) was the single compound to show an excellent potency against all the tested strains, thus providing valuable insights about the antifungal potential of this class of compounds and the structure-activity relationships.

## 1. Introduction

Aurantosides and rubrosides are orange-red pigments isolated from sponges of the genera *Theonella*, *Homophymia* and *Siliquariaspongia*, all belonging to the order Lithistida [1,2,3,4,5]. These complex molecules include three different structural moieties: a mono- or dichlorinated long (C_18_ to C_28_) conjugated polyene chain (terminating with an oxygenated five-membered ring in the rubroside subfamily), a tetramate ring, and an *N*-glycosidic moiety made up by one to three monosaccharide units, which are, invariably, pentose or deoxypentose sugars.

Compared to non-ribosomial cyclic and linear peptides and to polyketide macrolides, the most widely investigated secondary metabolites from sponges of the Lithistida order, aurantosides have been largely overlooked, also in terms of their biomedical potential. The reasons for this observation can be mostly found in the difficulties associated to their isolation in the pure form and to their relative instability, which is obviously related to the presence of the polyene moiety.

**Scheme 1:**
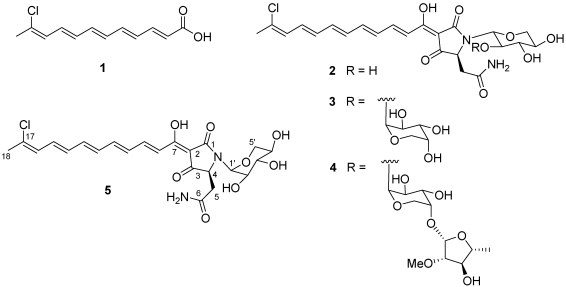


In the frame of our ongoing investigation of Indonesian invertebrates for bioactive secondary metabolites [6,7,8], we have recently analyzed a specimen of *Theonella swinhoei* (Theonellidae, Lithistida) and reported the isolation of dehydroconicasterol and of aurantoic acid (**1**) [9]. This latter compound closely resembles the polyene moiety of aurantosides G–I [4], although the biosynthetic relationship between aurantoic acid and aurantosides has not been established. Chemical analysis of the most polar fractions of the organic extract obtained from the same organism revealed that they were practically devoid of polar polypeptides or macrolides, while they contained consistent amounts of aurantosides. In this paper we describe the isolation and the structure elucidation of the three known aurantosides G–I (**2**–**4**) and of the new aurantoside J (**5**) from these fractions (see Scheme 1). In addition, we report the results of a detailed investigation of the antifungal activity of the isolated aurantosides, which revealed a strict dependency from the glycosylation pattern. 

## 2. Results and Discussion

A specimen of the sponge *T. swinhoei* was collected by hand in the area of the Bunaken Marine Park of Manado (North Sulawesi, Indonesia) and kept frozen until sequentially extracted with MeOH and CH_2_Cl_2_ by soaking the sliced sponge tissues (560 g wet wt). The extracts were combined, concentrated and partitioned between EtOAc and H_2_O. Then, the water-soluble fraction was partitioned against *n*-BuOH and the so obtained polar organic extract (3.8 g) was chromatographed on a C_18_ reversed-phase silica gel column, using a gradient solvent system going from H_2_O/MeOH 7:3 to H_2_O/MeOH 1:9. Repeated RP18 HPLC purifications (H_2_O/CH_3_CN mixtures, with 0.05% TFA, as eluents) afforded aurantosides G (**2**, 25.5 mg), H (**3**, 12.8 mg), I (**4**, 15.6 mg) and the new aurantoside J (**5**, 8.3 mg) in the pure form. The structures of the known aurantosides G–I (**2**–**4**) were identified on the basis of the comparison of their spectral data with those reported in the literature [4]. It is worth noting that all the aurantosides isolated during this work showed the same length and constitution of the polyene side chain, while aurantosides with longer and/or dichlorinated polyene chain (as aurantosides A–F) were not detected in our sample.

Aurantoside J (**5**) was isolated as an orange-red amorphous solid. Its ESI mass spectrum (positive ions) showed pseudomolecular ion peaks at *m/z* 517 and 519 [M + Na]^+^ in the ratio of about 3:1, suggesting the presence of a chlorine atom. High resolution measurements (see Experimental Section) assigned the molecular formula C_23_H_27_ClN_2_O_8_ to **5**, in accordance with the presence of eleven unsaturation degrees. 

The ^1^H NMR spectrum (700 MHz, CD_3_OD) of **5** revealed the presence of a polyene unit (nine multiplets from δ_H_ 6.30 to 7.63), of an allylic methyl group (δ_H_ 2.21), and of a tetramic acid glycoside, classifying compound **5** as a metabolite of the aurantoside family. All the proton signals were associated to those of the directly attached carbon atoms through the 2D HSQC experiment and the so obtained sets of ^1^H and ^13^C NMR data of **5** evidenced a close resemblance with parallel data reported for aurantoside G (**2**) [4], which also shared the same molecular formula of **5**. 

Combined inspection of COSY and TOCSY spectra allowed a deconvolution of the proton signals within the polyene moiety and the consequent assignment of the proton and carbon resonances, as well as of proton-proton coupling constants, within this spin system. The obtained data appeared in excellent agreement with those reported for aurantoside G, suggesting that **2** and **5** should share this part of their structures, including the all-*trans* geometry of the disubstituted double bonds. The *Z* geometry of the terminal, chlorine-bearing double bond, was secured on the basis of the ROESY cross-peak between the methyl singlet at δ 2.21 (Me-18) and the doublet at δ 6.30 (H-16). 

**Figure 1:**
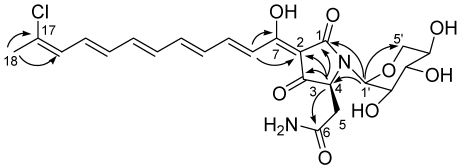
Key H→C HMBC correlations detected for aurantoside J.

The identification of the stereostructure of the tetramate moiety, as well as its attachment to the polyene chain, was largely based on the comparison with literature data. The HMBC spectrum (Figure 1) showed cross-peaks of the methine resonating at δ 4.18 (H-4) with C-1, C-2, C-3 and with the amide carbon at 174.1, while the doublet at δ 7.23 (H-8) showed cross-peaks with C-7 and C-2. This pattern of HMBC correlations and the consequent assignment of ^13^C NMR resonances were in perfect agreement with values reported for other aurantosides. In accordance with the behavior reported for aurantosides and for other tetramic acid derivatives [10], the keto-enol tautomerization of the adjoining tetramic acid portion of the molecule caused the appearance of a couple of small methine resonances (ratio 1:3 compared to other methine signals) in the sp^2^ region of the ^1^H NMR spectrum of **5**, which are most likely attributable to H-8 and H-9 on the minor tautomer. 

The spin system of a pentose monosaccharide was easily identified from the COSY spectrum. The HMBC correlation of the anomeric proton (δ_H_ 5.01, δ_C_ 82.9) with C-1 and C-4, as well as its relatively high-field resonance, defined its attachment to the nitrogen atom of the tetramate ring, while the HMBC correlation H-1′/C-5′ indicated its pyranose form. The large value of the ^3^*J* scalar coupling constants *J*_H-2′/H-3′_ and *J*_H-3′/H-4′_ (=10.2 Hz) and the small value of *J*_H-1′/H-2′_ (=2.6 Hz) allowed the identification of this sugar as a α-xylopyranose and strongly suggested the conformation depicted in Scheme 1. Of course, the predominance of different conformations in different solvents cannot be excluded. Given the small amount of compound available (largely used for evaluation of cytotoxic and antifungal activities, see below), the absolute configuration of the xylopyranosyl unit in **5** was not determined. However, a D configuration could be confidently assigned to this sugar, considering that the xylose units present in all the aurantosides isolated to date, including **2**–**4**, invariably proved to belong to the D series.

In conclusion, aurantoside J (**5**) is a new secondary metabolite whose structure differs from that of the known aurantoside G (**2**) for the configuration at the anomeric carbon C-1′ of the xylose unit. In this regard, it is worth noting that aurantoside J represents the first member of this class of compounds to show an *N*-glycosidic linkage with the α-configuration. 

All the members of the aurantoside family had been evaluated for their cytotoxic activity and data available in the literature [1,2,3,4] clearly suggest a close relationship between the length of the polyene chain and the toxicity against human cell lines: an increase in the length of the chain is associated to an increase of the cytotoxic activity. Accordingly, aurantosides G–I (C_18_) and aurantosides A–B (C_20_) are not cytotoxic, aurantosides D and E (C_22_) are moderately cytotoxic, while aurantoside F (C_24_) is strongly cytotoxic. In perfect agreement with this trend, the new aurantoside J (**5**) proved to be devoid of any cytotoxic activity (IC_50_ > 70 μM) against several human cell lines (data not shown).

Since the antifungal potential of aurantosides has remained practically undisclosed (with the single exception of the good antifungal activity against *Aspergillus fumigatus* reported for aurantosides A and B^3^), and given the increasing need for new active agents to face fungal infections, we decided to evaluate aurantosides G–J (**2**–**5**) for their antifungal activity against *Fusarium solani* and four *Candida* species. These strains have been selected since, together with *Zygomycetes*, they represent the main responsible of invasive fungal infections (IFIs), an increasingly important clinical dilemma, which engenders high rates of morbidity and mortality, particularly in immuno-compromised populations. In the management of patients with haematological malignancies or transplant recipients, an increase in the incidence of fungal diseases due to opportunistic infections of genera *Candida* and *Fusarium* is commonly observed [11]. Unfortunately, despite an antifungal treatment with available agents, the mortality rate of patients with IFIs remains extremely high. *Candida* infections are the most frequent cause of IFIs worldwide and, in USA, *Candida* species are the fourth most common cause of nosocomial blood stream infection [12]. Among the 50 species of *Fusarium* identified to date, *F. solani* is responsible of 50% of pathogenic infections in humans [13], which are life-threatening in immunocompromised hosts: recognized risk factors are skin lesions, use of corticosteroids, prolonged neutropenia and hematological malignancy [14]. 

The antifungal activity of aurantosides G–J (**2**–**5**) has been expressed as the Minimum Inhibitory Concentrations (MICs) and Minimum Fungicidal Concentrations (MFCs) for 90% and 50% of isolates. The MICs and MFCs of the four aurantosides against 15 clinical isolates of fungi (12 isolates for the four *Candida* species *C. albicans*, *C. parapsilosis*, *C. glabrata*, *C. tropicalis* and 3 isolates for *F. solani*) are shown in Table 1.

**Table 1:** *In* *vitro* activities of Aurantosides G–J against *Candida* spp. and *F. solani* ^a^.

Isolate ^b^	Aurantoside G MIC_50_/MIC_90_ (MFC_50_/MFC_90_) in µg/mL	Aurantoside H MIC_50_/MIC_90_ (MFC_50_/MFC_90_) in µg/mL	Aurantoside I MIC_50_/MIC_90_ (MFC_50_/MFC_90_) in µg/mL	Aurantoside J MIC_50_/MIC_90_ (MFC_50_/MFC_90_) in µg/mL
*C. albicans*	4/8 (4/8)	>16/>16 (>16/>16)	0.25/0.5 (0.25/0.5)	>16/>16 (>16/>16)
*C. parapsiolsis*	2/4 (4/4)	>16/>16 (>16/>16)	0.5/0.5 (0.5/0.5)	>16/>16 (>16/>16)
*C. glabrata*	4/8 (8/8)	>16/>16 (>16/>16)	0.125/0.125 (0.125/0.125)	>16/>16 (>16/>16)
*C tropicalis*	2/4 (2/4)	>16/>16 (>16/>16)	0.25/0.5 (0.5/0.5)	>16/>16 (>16/>16)
*F. solani*	16/>16 (16/>16)	>16/>16 (>16/>16)	1/2 (2/2)	>16/>16 (>16/>16)

^a^ By the CLSI M27-A2 and M38-A procedure; ^b^ Three isolates of each species were tested.

Only aurantosides G (**2**) and I (**4**) showed a detectable antifungal activity, but the activity of aurantoside G was only moderate-poor, with a MIC_90_ of 4–16 μg/mL against all the strains. In contrast, aurantoside I exhibited a very good antifungal effect against all the tested strains, especially against *C. albicans*, *C. glabrata* and *C. tropicalis*. The activity of aurantoside I against *F. solani* is also remarkable, given the high pathogenicity of this strain and the need of potent inhibitory agents. For the majority of isolates, MFCs of the test compounds were either the same as or one to two dilutions higher than the MICs.

The mechanism of the antifungal action of aurantoside I (**4**) has not been investigated; however, it could be related to the mechanism of other polyene antifungal agents as nystatin. Since all the compounds tested in this study are homogeneous in terms of the C_18_ polyene chain, the role of the sugar moiety in the modulation of the activity can be highlighted. Clearly, a trisaccharide moiety, present in aurantoside I and in the previously reported aurantosides A–B, is needed for the antifungal activity of this class of compounds. 

## 3. Experimental Section

### 3.1. General Experimental Procedures

Optical rotations (CHCl_3_) were measured at 589 nm on a P2000 Jasco polarimeter using a 10 cm microcell. ^1^H (700 MHz) and ^13^C (175 MHz) NMR spectra were measured on a Varian INOVA spectrometer. Chemical shifts were referenced to the residual solvent signal (CD_3_OD: δ_H_ 3.34, δ_C_ 49.0).Homonuclear ^1^H connectivities were determined by the COSY experiment; one-bond heteronuclear ^1^H-^13^C connectivities by the HSQC experiment; two- and three-bond ^1^H-^13^C connectivities by gradient-HMBC experiments optimized for a ^2,3^*J* of 8 Hz. Low- and high-resolution ESIMS spectra were obtained on a LTQ OrbitrapXL (Thermo Scientific) mass spectrometer. Medium pressure liquid chromatography was performed on a Büchi apparatus using a silica gel (230–400 mesh) column; HPLC was achieved on a Knauer apparatus equipped with a refractive index detector and analytical LUNA (Phenomenex) RP18 (250 × 4 mm) columns.

### 3.2. Animal Material, Extraction and Isolation

A specimen of *Theonella* *swinhoei* (order Lithistida, family Theonellidae) was collected in January 2008 along the coasts of the Bunaken Island in the Bunaken Marine Park (North Sulawesi, Indonesia).The species is very common in this area, from 20 to 50 m depth, on substrata subjected to strong currents. *In vivo*, the sponge is red-orange but specimens living in shadow habitats are pale pink to white. A voucher sample has been deposited at the Dipartimento di Chimica delle Sostanze Naturali, Università di Napoli Federico II (Man08-02). After homogenization, the organism (wet weight 560.2 g) was exhaustively extracted, in sequence, with MeOH and CH_2_Cl_2_. The combined extracts were partitioned between H_2_O and EtOAc and, then, the water-soluble fraction was partitioned against *n*-BuOH. The obtained polar organic extract (3.8 g) was subjected to medium pressure liquid chromatography over a C_18_ reversed-phase silica gel column, using a gradient solvent system going from H_2_O/MeOH 7:3 to H_2_O/MeOH 1:9. Fractions eluted with H_2_O/MeOH 6:4 and 1:1 were combined and subjected to HPLC purification on RP18 column (H_2_O/CH_3_CN 65:35, with 0.05% TFA, as eluent) to yield aurantoside I (**4**, 15.6 mg) in the pure form. Fractions eluted with H_2_O/MeOH 4:6 were combined and subjected to HPLC purification on RP18 column (H_2_O/CH_3_CN 35:65, with 0.05% TFA, as eluent) to yield aurantoside H (**3**, 12.8 mg) in the pure form. Fractions eluted with H_2_O/MeOH 3:7 and 2:8 were combined and subjected to HPLC purification on RP18 column (H_2_O/CH_3_CN 1:1, with 0.05% TFA, as eluent) to yield aurantoside G (**2**, 25.5 mg) and the new aurantoside J (**5**, 8.3 mg) in the pure form.

### 3.3. Aurantoside J *(**5**)*

Orange amorphous solid; [α]_D_^25^ −122 (*c* 0.1 in MeOH); ^1^H NMR (CD_3_OD, 700 MHz): δ_H_ 7.63 (dd, *J* = 15.3, 11.0 Hz, H-9), 7.23 (d, *J* = 15.3 Hz, H-8), 6.87 (dd, *J* = 15.0, 11.0 Hz, H-11), 6.68 (dd, *J* = 15.0, 11.0 Hz, H-15), 6.64 (dd, *J* = 15.0, 11.0 Hz, H-13), 6.55 (dd, *J* = 15.0, 11.0 Hz, H-10), 6.48 (overlapped, H-12, H-14), 6.30 (d, *J* = 11.0 Hz, H-16), 5.01 (d, *J* = 2.6 Hz, H-1′), 4.18 (bs, H-4), 2.78 (m, H-5a), 3.83 (dd, *J* = 11.5, 4.5 Hz, H-5′a), 3.77 (dd, *J* = 10.2, 2.6 Hz, H-2′), 3.58 (m, H-4′), 3.36 (t, *J* = 10.2 Hz, H-3′), 3.22 (dd, *J* = 11.5, 9.5 Hz, H-5′b), 2.68 (m, H-5b), 2.21 (s, Me-18); ^13^C NMR (CD_3_OD, 175 MHz): δ 201.7 (s, C-3), 175.7 (s, C-1), 175.2 (s, C-7), 174.1 (s, C-6), 145.5 (d, C-11), 144.4 (d, C-9), 141.0 (d, C-13), 134.5 (d, C-12), 134.3 (d, C-14), 134.0 (s, C-17), 133.0 (d, C-10), 132.3 (d, C-15), 126.7 (d, C-16), 121.5 (d, C-8), 101.5 (s, C-2), 82.9 (d, C-1′),77.8 (d, C-3′), 70.2 (d, C-2′), 69.6 (d, C-4′), 67.7 (t, C-5′), 62.5 (d, C-4), 38.0 (t, C-5), 26.8 (q, C-18). (+) ESI-MS *m/z* 517 and 519 (3:1) [M + Na]^+^. HR-ESIMS *m/z* 517.1350 (calcd for C_23_H_27_^35^ClN_2_O_8_Na 517.1354). 

### 3.4. Antifungal Activity

Test isolates were taken from the culture collection at the Operative Unit of Microbiology, Second University of Naples and included *Candida albicans*, *C. parapsilosis*, *C. glabrata*, *C. tropicalis* and *Fusarium solani*. *Candida* isolates were identified by using using standardized morphological and biochemical methods (tube germination, chlamydospora production and API20C) (bioMérieux), while *F. solani* was identified by colonial and microscopic morphologies and stored in sterile distilled water at 25 °C. The test isolates were subcultured from stocks in Sabouraud dextrose agar (SDA) (Oxoid) and incubated at 30 °C for 48 h for the *Candida* spp. and 72 h for *F. solani*. Colonies from cultures of *Candida* species were suspended in 6 mL of phosphate-buffered saline (PBS). The resulting suspension was adjusted to the turbidity of a 0.5 McFarland standard at a wavelength of 530 nm. To collect conidia following incubation, conidia of the *Fusarium* were harvested and hyphae were removed by filtration through sterile gauze. Conidia were washed with PBS and the suspension was adjusted to the turbidity of a 0.15–0.17 McFarland standard at a wavelength of 530 nm. The inoculum for the reference macrodilution method was made by a 1:100 dilution in sterile water; this was followed by a 1:20 dilution with RPMI-1640 medium (with glutamine, without bicarbonate, and with phenol red as a pH indicator) (Sigma) supplemented with 0.2% glucose and buffered to a pH of 7.0 with 0.165 mol/L MOPS (3-[*N*-morpholino] propanesulfonic acid) (Sigma). This resulted in a final inoculum of 0.5 × 10^3^ to 2.5 × 10^3^ CFU/mL. In each case, the inoculum size was verified by colony counts.

Stock solutions of aurantosides G–J (2–5) were prepared in sterile de-ionized water. Trays containing a 0.1 mL aliquot of the appropriate drug solution (2× final concentration) in each well were stored at −70 °C until use. The final concentrations of drug in the wells ranged from 0.008 μg/mL to 16 μg/mL. The minimal inhibitory concentrations (MICs) were determined in round-bottom 96-well microtiter plates using standard broth microdilution protocols described in the CLSI M27-A2 (for yeasts) or M38-A (for filamentous fungi) documents. The final inoculum was verified by plating 100 μL of the adjusted inoculum onto SDA. Plates were incubated at 30 °C and examined daily for fungal colonies for up to 48 h for *Candida* spp and up to 72 h for *F. solani*. Two drug-free controls were included in each test run: one contained medium alone (sterile control), and the other contained medium plus the inoculum (growth control). Each isolate was tested in duplicate in two independent experiments. The parameters MIC 50% and MIC 90% (concentrations able to inhibit 50% and 90% of the isolates, respectively) were also determined. 

The minimum fungicidal concentrations (MFCs) were determined for the test compounds only. After the MIC was read, 30 μL aliquots of suspension from each well showing total inhibition of visible growth were plated onto SDA and incubated at 30 °C. MFCs were read after 48 h (for *Candida* spp.) or for up to 72 h (for *F. solani*). The MFC was defined as the lowest drug concentration at which no colonies were observed. Each isolate was tested in duplicate in two independent experiments. The data were compared using Student’s *t*-test, and *P*-values < 0.05 were considered significant. 

## 4. Conclusions

In conclusion, the chemical investigation of an Indonesian specimen of *Theonella swinhoei* has yielded four aurantosides (G–J), one of which, aurantoside J (**5**), is a new compound exhibiting the unprecedented *N*-α-glycosidic linkage between the pentose and the tetramate units. The four isolated aurantosides have been tested against five fungal strains (four *Candida* and one *Fusarium*) responsible of invasive infections in immuno-compromised patients. The non-cytotoxic aurantoside I (**4**) was the sole compound to show an excellent potency against all the tested strains, thus disclosing two essential requirements to optimize the antifungal activity in this class of compounds: (i) a C_18_- to C_20_-polyene chain (to minimize the cytotoxic activity against human cell lines); and (ii) a trisaccharide chain attached at the tetramate ring. 
